# A myriad of methods to determine temporal summation of pain in people with musculoskeletal pain and healthy participants: a scoping review

**DOI:** 10.1097/PR9.0000000000001176

**Published:** 2024-09-04

**Authors:** Sjoerd C. Kielstra, Roland R. Reezigt, Michel W. Coppieters, Ralph de Vries, Lars Arendt-Nielsen, Kristian K. Petersen, David Yarnitsky, Gwendolyne G.M. Scholten-Peeters

**Affiliations:** a Department of Human Movement Sciences, Faculty of Behavioural and Movement Sciences, Amsterdam Movement Sciences Program Musculoskeletal Health, Vrije Universiteit Amsterdam, Amsterdam, the Netherlands; b Academy of Health, Department of Physical Therapy, Hanze University of Applied Sciences, Groningen, the Netherlands; c School of Health Sciences and Social Work, and Menzies Health Institute Queensland, Griffith University, Brisbane & Gold Coast, Australia; d Medical Library, Vrije Universiteit Amsterdam, Amsterdam, the Netherlands; e Center for Neuroplasticity and Pain (CNAP), Department of Health Science and Technology, School of Medicine, Aalborg University, Aalborg, Denmark; f Department of Gastroenterology & Hepatology, Mech-Sense, Clinical Institute, Aalborg University Hospital, Aalborg, Denmark; g Steno Diabetes Center North Denmark, Clinical Institute, Aalborg University Hospital, Aalborg, Denmark; h Department of Neurology, Rambam Medical Center, Haifa, Israel; i Laboratory of Clinical Neurophysiology, Technion Faculty of Medicine, Haifa, Israel

**Keywords:** Temporal summation of pain, Wind-up ratio, Quantitative sensory testing, Pain modulation, Pain measurement, Pain perception

## Abstract

Temporal summation of pain (TSP) is a human proxy for wind-up of dorsal horn neurons as assessed in animals. The common paradigm for eliciting TSP is evoked by repetitive nociceptive stimuli of equal intensity. Various stimulation and assessment protocols have been used. This scoping review aims to provide insight into key elements of TSP stimulation and assessment: modality, instruments, test location, familiarization, train characteristics, and calculations. PubMed, Embase, and Ebsco/CINAHL were searched for studies that measured TSP in adults with musculoskeletal conditions and healthy people. Four hundred six studies were included. Mechanical stimuli were the most commonly used modality (250 studies), followed by thermal stimuli (125 studies). Forty-six different instruments were used. Disregarding studies on widespread musculoskeletal pain and healthy participants, 40 studies evaluated TSP at painful sites, 77 in remote areas, and 66 in both locations. Of the 13 tested locations in patients, the hand (74 studies), lower leg (64 studies), and forearm (59 studies) were most commonly tested. A single practice round was the most common familiarization method (46 studies). Repeated stimuli were applied using 31 different frequencies (0.03-200 Hz) and sustained stimulations ranging from 5 to 1080 seconds were used. Twenty-two different train lengths, 63 different calculations (37 absolute, 19 relative, and 7 alternatives using data directly), and 14 different outcome measures (eg, self-reported pain rating scales and reflex thresholds) were used. Temporal summation of pain protocols vary excessively, hindering the comparison and pooling of results. None of the studies provided substantiation for their protocol choice.

## Abstract 2

Supplemental Digital Content is Available in the Text.

## 1. Introduction

Somatosensory and pain sensitivities can be assessed using a battery of noninvasive standardized quantitative sensory tests.^25,26^ Quantitative sensory test can be divided into static and dynamic tests.^20^ Static tests refer to mechanical or thermal detection thresholds (eg, vibration, warmth, and cold detection), pain thresholds, and pain tolerance testing. Dynamic tests include temporal summation of pain (TSP) and conditioned pain modulation.

Conditioned pain modulation is a paradigm designed as a tool to assess the balance between descending inhibitory and facilitatory pathways in humans^2,7,11^ and is based on diffuse noxious inhibitory control concept developed for animal research.^6,27^ Temporal summation of pain is used as a proxy to assess the summation and hence increased pain after repetitive stimuli of equal stimulus intensity.^4,23^ Temporal summation of pain is based on the neuronal electrophysical wind-up principle described in animals, as measured by direct neuronal recordings or reflexes.^16^ Originally wind-up was identified as an increase in activity in dorsal horn cells after repetitive stimulation of C-fibers with an electrical current at a frequency of 0.3 to 5 Hz.^16,24^ In humans, TSP assesses facilitatory pathways by the perception of increased pain intensity, increased nociceptive reflexes, or evoked potentials after repetitive nociceptive stimuli of equal stimulus intensity.^4,15,23,25,26^

Diverse protocols have been used to assess TSP in healthy participants and patients with various conditions.^3,7,9,21,23,28^ Temporal summation of pain is assessed by applying a train of nociceptive stimuli, but different modalities, instruments, train characteristics, calculations, and test locations hinder the comparison of TSP outcomes between studies. In contrast to recommendations for the assessment and reporting of conditioned pain modulation,^32^ there are no recommendations for the assessment or reporting of TSP. Before such recommendations for TSP can be generated, descriptions of the existing methods are required. This scoping review aimed to map the different elements of TSP protocols to gain a better understanding into how TSP is measured and reported, which may pave the way for developing gold standards.

## 2. Methods

A scoping review was conducted to identify and map key TSP elements. The protocol was developed according to the scoping review methodology framework.^5,18^ The Preferred Reporting Items for Systematic Reviews and Meta-Analyses extension for scoping reviews checklist was followed for reporting.^29^ The protocol was registered at the Open Science Framework (10.17605/OSF.IO/J2TNP).

### 2.1. Eligibility criteria

Because of the large body of research on TSP, this scoping review focused on studies that assessed TSP in people with musculoskeletal conditions, as defined by the global burden of disease studies and the World Health Organization,^12,13,31^ irrespective of the inclusion of healthy controls, and studies that only assessed healthy participants. Original studies were eligible if at least 4 of the 5 key elements of TSP measurement were reported (modality, instrument, test location, train characteristics, and calculation). No language restrictions were applied. Pharmacological interventions, reviews, conference papers, and editorials were excluded.

### 2.2. Information sources and search strategy

In line with the guidelines for scoping reviews, our search consisted of 3 phases: (1) a limited initial explorative search, (2) a full search, and (3) a search of the reference lists of the included articles.^5^ The initial search consisted of a limited search in 1 database (PubMed). In this phase, articles were screened, keywords were identified, and the strategy for the full search was created in collaboration with a medical research librarian (R.V.).

Systematic searches were conducted in the bibliographic databases like PubMed, Embase, and Ebsco/CINAHL from inception until February 14, 2023. References of the identified articles were searched for additional relevant publications. Duplicate articles were removed. The search strategy for PubMed can be found in Appendix 1 (available at http://links.lww.com/PR9/A237).

### 2.3. Selection of sources of evidence

All identified citations were uploaded in Rayyan, a web and mobile app for systematic and scoping reviews.^22^ First, a random sample of 15 abstracts were successfully pilot-tested by 2 reviewers (S.C.K. and R.R.R.) to address possible discrepancies in applying the selection criteria. Second, these 2 reviewers independently assessed the titles and abstracts identified in the full search for eligibility using predefined inclusion and exclusion criteria. Subsequently, the 2 reviewers independently screened the full texts of the selected articles. Discrepancies were resolved by discussion, or when no agreement could be reached, a third reviewer (G.G.M.S.-P.) was consulted to help resolve the discrepancy.

### 2.4. Data charting process and data items

Data extraction was independently performed by 2 reviewers (S.C.K. and R.R.R.). The following data were extracted and stored in Microsoft Excel: study characteristics (eg, first author name, year of publication, country of origin, study population, and sample size) and key elements of TSP, such as modality, instrument, test location, familiarization, train characteristics (eg, frequency, train length, time between trains, and number of trains), and calculations, using an agreed template. The variables to be extracted were predefined by 3 investigators (S.C.K., R.R.R., G.G.M.S.-P.), and the data extraction tool was pilot-tested in 3 articles that were not included in the scoping review. A distinction was made between “healthy controls” and “healthy participants.” Healthy controls were defined as those who acted as the healthy control group in a study that included participants with musculoskeletal conditions. In contrast, the term “healthy participants” was reserved for when a study only included healthy participants. This distinction is important, as for healthy controls, elements of the protocol (such as test location) were determined by the studied patient population (eg, low back pain), whereas, for healthy participants, such considerations did not apply.

### 2.5. Data synthesis

The extracted data were summarized and grouped according to the TSP key elements. Sankey diagrams were used to graphically represent the relationship between key elements with modality as the grouping variable.

## 3. Results

**Figure 1.**
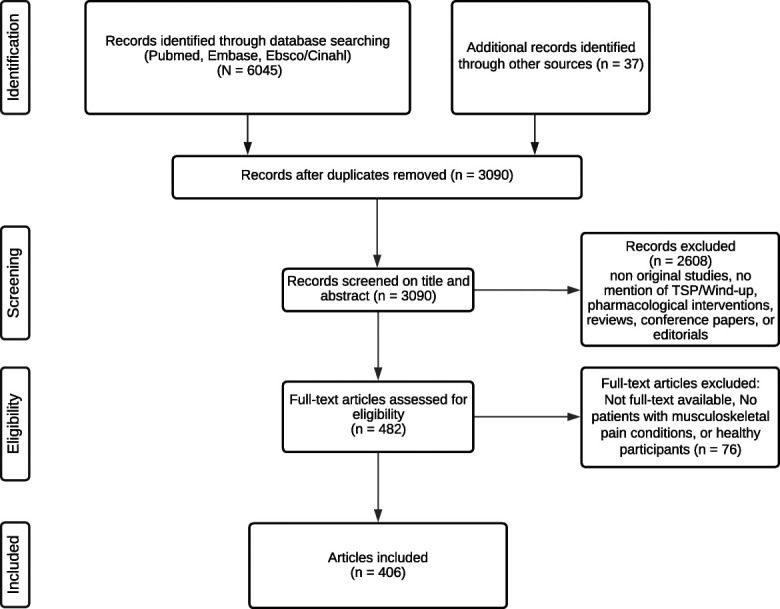
Flow diagram of the search strategy. Results for studies on TSP and musculoskeletal conditions. TSP, temporal summation of pain.

The literature search yielded 6082 results. After duplicate removal, 3090 unique articles were identified, of which 2608 were excluded after title/abstract screening. The remaining 482 full-text articles were retrieved and assessed for their eligibility. After full-text screening, 75 articles were excluded based on the selection criteria. Subsequently, data from 406 unique articles were extracted and synthesized (Fig. 1). Please see Appendix 3 (available at http://links.lww.com/PR9/A237) for the details per study. Appendix 2 (available at http://links.lww.com/PR9/A237) is the reference list of the included studies in Appendix 3 (available at http://links.lww.com/PR9/A237).

Because several studies contained more than 1 variation for a key element (eg, mechanical and thermal stimulation were used), the number of studies/participants presented in the Sankey diagrams can be higher than the number of included studies/participants. For example, participants from 1 study who were measured by thermal and mechanical stimulus modalities are presented in the Sankey diagram for both modalities.

### 3.1. Participants and conditions

Across the included studies, TSP was assessed in 30,909 patients with musculoskeletal conditions and 20,590 healthy controls/participants. Temporal summation of pain was assessed in 38 musculoskeletal conditions in 225 studies, and 182 studies included only healthy participants. Knee osteoarthritis was the most frequently studied condition (50 studies), followed by chronic low back pain (27 studies) (Appendix 3 [available at http://links.lww.com/PR9/A237]).

### 3.2. Modalities and instruments

Five stimulus modalities were used to measure TSP: 250 studies used a mechanical modality, 125 studies used a thermal modality, 42 studies used an electrical modality, 3 studies used a chemical modality, and 2 studies used an ultrasonic modality. Sixteen studies used more than 1 modality.

Forty-six different instruments were used to measure TSP. These instruments were grouped into 8 classes. Instruments for mechanical stimulation were categorized into 3 subclasses based on contact area: small contact area (n = 137) (eg, pinprick), medium contact area (n = 68) (eg, pressure algometer), and large contact area (n = 49) (eg, pressure cuff). Thermal stimulation was categorized into 2 classes: small contact area (n = 123) (eg, thermal stimulator) and large contact area (n = 3) (eg, water bath). Electrical stimulation was achieved using a constant-current stimulator. Chemical stimulation involved the injection of glutamate or hypertonic saline, and ultrasound was assessed using a custom-built setup. Eight studies did not report the instruments used.

### 3.3. Measurement location

Temporal summation of pain was assessed at 13 different locations. When disregarding studies on widespread pain, among the 224 studies involving patients with localised pain states, the most common locations were the hand (74 studies), lower leg (64 studies), and forearm (59 studies). Furthermore, TSP was measured locally (within the painful area) in 40 studies, remotely in 77 studies, and in painful and remote areas in 66 studies. The forearm was used as a remote site in 26 studies, the hand in 4 studies, and the lower leg in 42 studies. The most painful body site was assessed in 3 studies, allowing variation in location between participants. One study did not report the location. In the 182 studies that included healthy participants, the hand (60 studies) was the most common area, followed by the forearm (58 studies) and lower leg (46 studies).

### 3.4. Familiarization

Familiarization procedures were either not reported or not performed in the majority of studies (310 studies). When reported, a single practice round was the most common method (46 studies). Some studies mentioned familiarization without providing further details (18 studies).

### 3.5. Stimulus and train characteristics

Stimulus intensities could be divided into predetermined intensities across all participants (ie, the same intensity for all participants; 165 studies) and individualized pain intensities (235 studies). In 117 studies, the individual pain threshold was used as the single stimulus intensity, other studies used a predetermined suprathreshold percentage, pain detection threshold, or used a marker such as “sense of discomfort, slightly painful, or unpleasant.” The stimulus intensity was unclear or not reported in 7 studies.

Repeated stimulation (361 studies) was used more often than sustained stimulation (34 studies). Thirty-one different frequencies of repeated stimuli were used, ranging from 0.03 Hz^14^ to 200 Hz.^8^ The most commonly used frequency was 1 Hz (206 studies). Twelve studies did not report the frequency used.

Twenty-two different train lengths were identified, and the number of repetitions in the train of stimuli ranged from 2 to 500. The most commonly used train length was 10 stimuli (252 studies). Eleven different durations for a sustained stimulus were used, ranging from 5 to 1080 seconds. The most commonly used sustained stimulus durations were 600 seconds (9 studies) and 120 seconds (10 studies).

**Figure 2.**
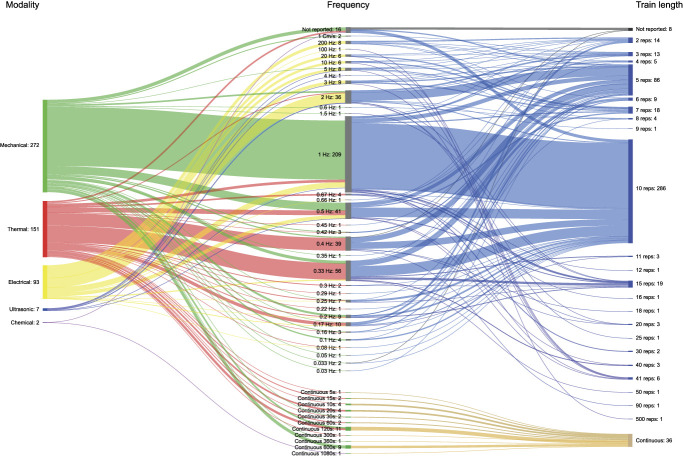
Modality, frequency, and train length. Shankey diagram showing the link between the number of TSP studies per modality, frequency, and train length used to measure TSP. Continuous, sustained-stimulation; Reps, repetitions. The node and path sizes linked to each modality, frequency, and train length were scaled to allow direct comparison within the figure. TSP, temporal summation of pain.

The number of trains varied from 1 to 16 stimuli trains. A single-train application was most commonly used (132 studies), followed by the application of 3 trains (52 studies). However, the number of trains was often not reported (127 studies). Figure 2 shows the frequencies and repetitions used in these studies.

### 3.6. Calculations

**Figure 3.**
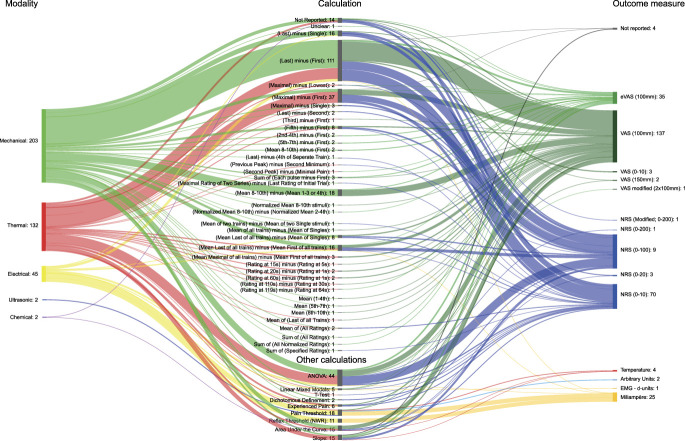
Absolute calculations and statistical comparisons. Shankey diagram showing the link between the number of studies per modality, absolute calculation, statistical comparisons, no calculations, and the type of outcome measure used to measure TSP. The description of the calculations was shortened to improve the visual presentation. For example, “[last] minus [first]” stands for “the intensity following the first stimulus is subtracted from the intensity of the last stimulus of the train.” The node and path sizes linked to the modality, calculation, statistical comparisons, and outcome measures are scaled and matched with those of Figure 4 to allow for direct comparison. TSP, temporal summation of pain.

**Figure 4.**
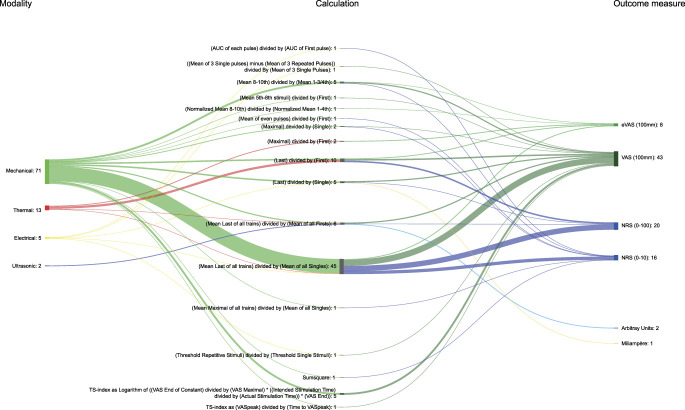
Relative calculations. Shankey diagram showing the link between modalities, relative calculation, statistical comparisons, no calculations, and outcome measures used to measure TSP. The different calculations have been shortened to give a better visual presentation, for example: “[mean last of all trains] divided by [mean of all singles]” stands for “the mean of the last stimuli of every train is divided by the mean of all singles that were assessed before each train.” AUC, area under the curve; eVAS, electronic visual analog scale; NRS, numeric rating scale; TSP, temporal summation of pain; VAS, visual analog scale. Node and path sizes linked to modality, calculations, no calculations, and outcome measures are scaled and match those of Figure 4 to allow for direct comparison.

Sixty-three different variations were used to quantify the TSP effect (Figs. 3 and 4). We created 2 groups to provide an overview: (1) absolute effect, meaning a calculation that uses the distance between the baseline and the selected measurement (eg, subtraction) to obtain a TSP outcome, and (2) relative effect, meaning a calculation where the selected measurement is divided by the baseline to obtain a TSP outcome. There were 37 ways to calculate the absolute TSP effect. The most frequently used calculation was pain intensity at the last stimulus of the train minus pain intensity at the first stimulus of the train (107 studies). We found 19 variations for calculating the relative TSP effect. The most common calculation for the relative effect was mean pain intensity of the last stimulus of all trains divided by the mean pain intensity of each single stimulus of all trains (43 studies).

As relative calculations cannot be performed when the single or first stimulus (depending on the calculation used) is pain-free (eg, visual analog scale [VAS] or numeric rating scale [NRS]: 0/10), multiple methods were described to circumvent the problem of dividing by 0. Six studies excluded cases with a baseline score of 0, and 6 other studies used Bartlett method of adding a small constant (eg, 0.01) to each rating. More commonly used was the individualization of the stimulus (240 studies). Individualization was achieved by identifying a specific (pain) intensity per participant and using that intensity for the stimulus. In 33 studies, no information on handling the 0 score was reported, even though a relative calculation was used.

Seven calculations other than absolute or relative calculations were identified for determining TSP. The most frequently used method was a form of statistical comparison (eg, analysis of variance) (46 studies), followed by a threshold comparison (eg, reaching the threshold after a train of stimuli [25 studies]). Finally, 15 studies did not report the methods used to quantify TSP. Appendix 3 (available at http://links.lww.com/PR9/A237) lists the different calculation methods used to quantify TSP.

Fourteen different outcome measures were identified. Pain intensity was measured most commonly using a pain intensity scale, such as multiple variations of VAS (194 studies) and the NRS (188 studies). The most used VAS was a paper-based VAS with a scale of 0 to 100 mm (158 studies). Alternatively, the stimulus intensity necessary to reach a predetermined experience (eg, mA to achieve a predetermined pain or Nociceptive Withdrawal Reflex) was used in 26 studies. Figures 3 and 4 show the calculations and outcome measures.

## 4. Discussion

This scoping review revealed a large variety of protocols to assess TSP. Modalities were mechanical, thermal, electrical, chemical, or ultrasonic. The application area ranged from a small area (eg, pinprick) to stimulation of a larger body part (eg, submersion of a hand in water). TSP was typically evaluated using repeated stimuli, varying in frequency (0.03-200 Hz) and number of stimuli (2-500). However, sustained stimuli were also used, such as the cold pressor test. In patients with musculoskeletal conditions, TSP has been assessed in both painful and nonpainful areas. Observation of the TSP phenomenon in studies using vastly different protocols regarding stimulus modality, application area, and frequency suggests that TSP is a robust phenomenon. However, the disadvantage of multiple approaches is that it hinders the comparison and pooling of the findings from different studies.

None of the included articles justified nor directly addressed the methodology on how to measure TSP in musculoskeletal conditions or healthy people. Instead, it seems to be primarily influenced by the available tools within the respective settings and the established practices within each laboratory. Furthermore, no studies evaluated the effect of different key elements on the magnitude of the TSP phenomenon. This review focuses on musculoskeletal conditions that could be considered arbitrary; however, there is a need for more information on TSP methodology in this area. The location where to measure TSP in musculoskeletal conditions remains an area of uncertainty. Through the inclusion of musculoskeletal conditions and healthy participants, instead of focusing on a specific condition (eg, knee osteoarthritis), a comprehensive overview regarding TSP measurement locations relative to the painful area (eg, in the most painful area, close to the painful area, or remote of the painful area) could be documented.

Therefore, using the findings of this scoping review as a foundation, we are currently developing recommendations and reporting guidelines for the assessment and reporting of TSP. In the sections below, we briefly elaborate on some of the considerations to determine the elements of a TSP protocol.

### 4.1. Frequency

We observed the use of a wide range of frequencies. For electrical stimulation, TSP seems to be elicited at 0.3 Hz or higher, with maximum reflex facilitation beyond 10 Hz.^4^ Temporal summation of pain seems to be elicited by mechanical stimulation at 0.16 to 1.00 Hz. However, pain from low-frequency mechanical stimulation (∼0.16 Hz) may be because of peripheral sensitization rather than a central mechanism.^19^ With the use of a thermal modality, TSP seems to be elicited at 0.2 to 0.3 Hz or higher. There seems to be a lower limit of 0.3 Hz for TSP after preclinical work.^16,24^

### 4.2. Stimuli repetitions

The large range of repetitions within a train (2-500) can be explained by the interaction between modality, chosen instrument, frequency, and the number of trains. All these components are related and of importance when designing a protocol to evoke TSP.

### 4.3. Stimulus intensity

A nociceptive, repetitive stimulus activates both Aδ fibers and C fibers, causing TSP.^10,16^ In a state of allodynia, however, a nonpainful, non-nociceptive, repetitive stimulus can also cause summation through changed wide-dynamic-range connections and can elicit pain.^4,10^ When aiming to mimic wind-up at the dorsal horn with the paradigm of TSP, the intensity of the initial stimulus is likely important (ie, the initial stimulus should be nociceptive). Even though nociceptive stimuli do not always elicit pain, pain is important in the clinical context as acute pain perception arises from nociceptive stimuli. However, although activation of C fibers typically correlates with pain perception after chemical and thermal stimulation, this is not consistently the case for mechanical stimulation.^30^ This may be because of simultaneous activation of A-beta mechanoreceptors, requiring high-intensity stimulation for pain perception.^30^ In addition, recent studies propose that thermal stimulation intensity at pain threshold +2°C is advocated to avoid floor effects while researching TSP.^17^ However, commencing stimulation at higher intensities constrains the potential for summation, given the finite nature of pain scales. This presents a challenge for researchers in clinical settings to prevent both floor and ceiling effects.

### 4.4. Calculation

Several methods have been used for TSP calculations (absolute, relative, statistical, and threshold methods). Each calculation has its specific characteristics that should be considered. The absolute calculation methods assume a pain rating scale to have interval or ratio scale properties (ie, equal intervals between values, for example between 20 and 40 and between 50 and 70 mm on a 0- to 100-mm VAS). Furthermore, high initial ratings limit the potential to increase the scoring because of using a finite scale, creating a ceiling effect that may affect the magnitude of the measurement of the TSP effect. When using relative calculation methods, the baseline score may skew the TSP effect; the closer the baseline score is to 0, the greater the TSP effect (eg, 4/2 = 2 vs 4/1 = 4). Moreover, when the baseline is 0 (ie, the initial stimulus is not painful), relative calculation cannot be performed. Although multiple solutions exist to avoid a baseline score of 0, all (except individualization) alter the results, making direct comparisons difficult. Statistical methods have the disadvantage of complexity because they require a complex process before incorporating outcome scores for analysis. Noteworthy, only 1 study directly compared the influence of 2 calculations on the TSP outcome (wind-up ratio vs sum square).^1^ This study showed that the sum square calculation was more sensitive in people with experimentally induced pain.^1^

### 4.5. Outcome measures

The comparison of TSP effects across different studies is hindered by the use of different outcome measures (eg, pain intensity measured with pain rating scales, nociceptive reflexes, and evoked potentials). However, pooling outcome measures (VAS and NRS) through transformation is possible.

## 5. Conclusion

There is variability between studies for each element of the measurement of TSP. The most commonly applied methods involve a mechanical modality with a small contact area (pinprick), 10 repetitions at 1 Hz, a fixed stimulus intensity (256 Nm), and an absolute calculation. Reasons for the popularity of this method may involve relatively low cost of the instrumentation, ease of delivery, and portability, making it feasible for both a laboratory and a clinical setting. However, despite its popularity, there is no evidence that the combination of 10 repetitions at 1 Hz, a fixed stimulus intensity (256 Nm), and an absolute calculation is superior to other methods to assess TSP. Considering the large variety of methods used to successfully elicit TSP, TSP seems a robust phenomenon. Moreover, as stated in the discussion, multiple questions can be raised regarding this paradigm. For example, a single stimulus with a 256-Nm weighted pinprick is painless in many people, questioning whether this initial stimulation is sufficiently nociceptive to stimulate the dorsal horn nociceptive neurons as intended with TSP. A nonpainful initial stimulus also compromises relative TSP calculations. This scoping review documented multiple possibilities and the variability in TSP measurement and reporting. This forms the basis for expert consultation and consensus recommendations on how to best evoke and quantify TSP, which is currently underway. The focus of this scoping review was limited to patients with and without musculoskeletal pain. Although the inclusion of studies on neuropathic pain, visceral pain, and other pain states would have been interesting, this would have resulted in an unmanageable volume of literature. Despite our rigorous efforts to avoid bias, potential selection bias cannot be ruled out when there is no mention or suggestion of TSP/wind-up in the title or abstract, or when incorrect inferences were made by the researchers based on the available information. It is possible that a small number of studies that met the selection criteria was not correctly identified. However, we believe that our conclusions regarding the myriad of methods to measure TSP would only be strengthened if additional studies would have been included.

## Disclosures

The authors have no conflicts of interest to declare.

## Supplemental digital content

Supplemental digital content associated with this article can be found online at http://links.lww.com/PR9/A237.
